# A chart review of management of ischemic stroke patients in Germany

**DOI:** 10.3402/jmahp.v3.24223

**Published:** 2015-09-24

**Authors:** Patrice Verpillat, Julie Dorey, Chantal Guilhaume-Goulant, Firas Dabbous, Julie Brunet, Samuel Aballéa

**Affiliations:** 1 H. Lundbeck A/S, Issy Les Moulineaux, France; 2 Creativ-Ceutical, Paris, France; 3 laboratoire ERIC, Université Lumière Lyon 2, Bron, France; 4 University of Illinois at Chicago, Chicago, IL, USA; 5 Hôpital de la conception PUI UF 3536 CIC, Marseille, France

**Keywords:** ischemic stroke, healthcare resource utilization, thrombolysis, long term management

## Abstract

**Background:**

Ischemic stroke (IS) poses physical, emotional, and economic burdens on both patients and the healthcare system in Germany. However, the management of IS is not well described, especially after hospital discharge. In this study, we aim to describe the management of IS at onset, admission, and during follow-up.

**Methods:**

German general practitioners (GPs) (*n=*40) extracted data on patient characteristics, hospitalizations, discharge, and ambulatory care from both GPs patient databases and hospital letters. Descriptive analyses were conducted.

**Results:**

The sample included 185 patients with a mean age of 70 years [standard deviation (SD)=11.7]. Most patients (63%) contacted the Emergency Medical Services, while 36% contacted their GPs. The majority of patients were hospitalized within 1 h from onset, and the length of stay was on average 14 days. Half of the patients (50%) were admitted to the stroke unit, and 16% of patients received thrombolysis treatment with 2 h (SD=2.6) of time to treatment. Of the admitted patients, 32% were discharged to their homes, while the remaining patients were discharged to nursing homes (16.2%) and rehabilitation centers (47.6%). During the 12 months follow-up, 22% of patients were re-hospitalized and patients visited their GP (11.7 times), psychologist or psychiatrist (9.5 times), and neurologist (2.2 times). Death rate after stroke event was 13%.

**Conclusion:**

The rate of patients who received thrombolysis is lower than the optimal rate in Germany. More research is needed to determine the factors that could predict the utilization of thrombolysis treatment.

In Germany, stroke affects 200,000 people annually and claims the lives of 66,000 people, making it the third cause of death. It is also the cause of 64,000 disabled patients annually with 15% of them requiring external assistance. The incidence stroke rate in Germany has been estimated to be approximately 160–240 per 100,000 people annually, with half of the patients being older than 70 years of age (1).

Beyond the physical and emotional burden, stroke inflicts a considerable economic burden on both payers and society. In 2004, the total financial burden on a major source of healthcare funding in Germany, the statutory health insurance (SHI), was estimated at €7.1 billion. This cost, in an incidence-based, bottom-up and direct-cost-of-ischemic-stroke study from the third-party payer's perspective, was mostly driven by outpatient treatment (40%; €2.8 billion), inpatient treatment (22%; €1.6 billion), rehabilitation (21%; €1.5 billion), and nursing (17%; €1.2 billion) (2). Further, the average cost for 12-day hospitalization during the acute phase was estimated to be €4,650 per patient (49% of direct costs) while total direct cost over 12 months was estimated at €9,452. From a societal perspective, the mean indirect costs were €2,014, with €1,344 attributed to days off work and €670 attributed to early retirement. Total associated stroke costs are impacted by patient characteristics, severity, and type of stroke (3).

Through information campaigns directed towards the general German population, prevention guidelines are well established in terms of raising awareness about stroke threats. The German Neurological Society (DGN) and the German Stroke Society (DSG) issued two joint guidelines entitled *Primary and Secondary Prevention of Cerebral Ischemia in 2008* and *Acute Treatment of Ischemic Stroke in 2009*. The primary objective was to prevent cerebral ischemia or transient ischemic attacks (TIAs) in patients without previous cerebrovascular diseases. The second aim was to prevent recurrent cerebral ischemia (TIA or stroke) after the initial event (4). These guidelines provided practical recommendations on different risk factors’ prevention, treatment, and suggestions for medication, but they lacked timelines for treatment duration. In addition to DSG and DGN, Germany also adopted the European Stroke Organization's (ESO) Guidelines for Management of IS and TIA in 2008.

Several studies have examined stroke management throughout both pre-hospital and acute phases. These studies evaluated interventions such as dissemination of knowledge about stroke to the general population, described mode of arrival, arrival time, and delays in arrival to the hospital and transfers to units within hospitals (5–8). Results from these studies highlighted the effectiveness of dissemination of stroke knowledge, so that patients, who received an educational letter describing stroke symptoms and the importance of calling the Emergency Medical Services (EMS) were admitted to facilities of care at a faster rate than those who did not receive the letter (5). Education, knowledge of the time window, direct contact with an emergency facility after stroke onset, number of known symptoms, living with someone, and having a stroke history were factors that improved arrival time (7).

The management of IS post-discharge is not well described in the literature, as there are only few studies describing patient pathways during the follow-up period. The CERISE project, which compared service delivery after discharge in four European rehabilitation units (SRU) at 2, 4, and 6 months, reported that Germany had the highest discharge-to-home rates compared to Switzerland, Belgium, and the UK (9). Yet another study, which evaluated the effectiveness of semi-intensive stroke unit (SI-SU) compared with conventional care (CC) for patients with acute IS or TIA over a 20-month period, estimated the average length of hospitalization to be 11.2 days in SI-SU and 13.3 in CC (10). Although these studies had a follow-up period, they did not report in detail the pathways IS patients had followed to manage the disease.

## Objectives

We sought to describe the management of IS at the pre-hospitalization phase, the acute phase (during hospitalization) as well as during 1 year of follow-up, relying on general practitioners (GPs) to extract all information about their IS patients.

## Methods

This is a retrospective chart review study, in which 40 GPs were randomly recruited from a representative panel of German GPs. The GPs were responsible for completing a questionnaire about their patients, relying on medical files. GPs were asked to include patients, who met the inclusion criteria, i.e., stroke patients, who did not suffer from transitional stroke, hemorrhagic stroke, or stroke with undetermined reasons. Patients were selected in a chronologic order based on stroke onset date (starting with most recent case) during 2007–2008. All data extraction took place between November 2009 and January 2010.

The questionnaire was designed to collect information by the GPs on IS patients with regards to: 1) patient characteristics (age, sex, occupation, living condition, medical history including stroke history, cardiovascular history, and previous ischemic event), 2) pre-hospitalization period (first health care contacts, mode of arrival), 3) hospitalization (type of hospital, first unit admitted to, units transferred to within hospital, imaging procedures, time between admission and treatment, type of treatment and length of stay in the different hospital units, severity scales), and 4) one-year follow-up after discharge (first place post-discharge, re-hospitalization, outpatient care, sick leave, and changing occupation). Information with regards to arrival method, type of hospital, first unit admitted to, units transferred to within hospital, imaging procedures (CT scans, MRI, ultrasound, or cerebral angiogram) and time between admission and treatment, type of treatment, and length of stay were documented in the hospital letter that was sent to the GPs from each treating hospital. Questionnaires were completed by GPs based on their electronic and paper medical records including hospital letters reporting the different units where patients stayed at hospital and a summary of the results of imaging tests. Means standard deviations, frequencies, and percentages are reported.

In an effort to validate data extractions, a random sample of five GPs was selected to confront study outcomes to GPs’ experience, to identify GPs’ level of insight on patient management in acute phase at hospital and post-discharge in rehabilitation centers, and to assess GPs’ opinions when confronting our study results to the available evidence in literature.

## Results

A sample of 40 GPs was randomly recruited, who extracted information for the 204 patients included in this study. Of these patients, 19 were excluded because complete 12 months of follow-up was not available.

The analyzed sample consisted of 185 patients, who were mostly retirees (74%) with a mean age of 70±11.7 years. Based on the interviews, GPs saw at least 10 stroke cases per year with average age ranging between 70 and 75 years. The sample included slightly more male patients (53%), and the majority was living in an urban setting (72%). Although the vast majority of this population did not have a prior stroke event, 73.5% of them had cardiovascular diseases or stroke risk factors (Table 1). Most patients (63%) contacted the EMS while a non-negligible number of patients first contacted their GP (36%). Most patients were transported to health care facilities via emergency vehicles (Table 2).

**Table 1:** Patient characteristics

Characteristic	Total, *N*=185 *n* (%)
Age mean (SD)	69.8 (11.8)
Males	98 (53)
Urban	133 (71.9)
Marital status	
Married/living with a partner	117 (63.2)
Single	68 (36.8)
Current place of living	
Home	57 (30.8)
Nursing home	19 (10.3)
Missing	102 (55.1)
Employed at the time of event	48 (25.9)
Medical conditions	
Ischemic event	16 (11.8)
Myocardial infarction	26 (19.1)
Angina pectoris	33 (24.3)
Carotid stenosis	29 (21.3)
Arteriopathy of lower limbs	22 (16.2)
Aortic aneurysm	2 (1.5)
Atrial fibrillation	35 (25.7)
Others	64 (47.1)

SD=standard deviation.

**Table 2:** Pre-hospitalization phase

	Total, *N*=185 *n* (%)
Contact with health professional at time of event	166 (89.7)
Type of health professional contacted at the time of event	
General practitioner (referee)	39 (23.5)
Emergency medicine physician	73 (44.0)
Emergency department	3 (1.8)
Other general practitioner	5 (3.0)
Other	1 (0.6)
Referee and emergency medicine physician	4 (2.4)
Referee and ambulance corps	11 (6.6)
Referee and other general practitioner and ambulance corps	1 (0.6)
Mode of arrival to Hospital	
Emergency ambulance	98 (57.0)
Individual car	19 (11.0)
Ambulance service	51 (29.7)
Other	2 (1.2)
No answer	2 (1.2)

### Hospitalization

Patients were admitted to specialized hospitals (34.9%), general hospitals (25.0%), basic general hospital (25%), and primary care (14%) (Table 3). Almost half of the patients were admitted directly to a stroke unit; other admission units varied between the emergency unit (12.8%), intensive care unit (16.3%), neurology unit (11.6%), and internal medicine unit (16.9%).

**Table 3:** Hospitalization and Healthcare resource utilization at acute phase

	Total, *N*=185 *N* (%)
Hospitalization	
Length of stay in Days^a^ (LOS) in hospital, mean (SD)	13.6 (9.8)
Time in hours between admission and treatment, mean (SD)	2.0 (2.64)
Type of Hospital	
Specialized hospital	60 (34.9)
General hospital	43 (25.0)
Basic general hospital	43 (25.0)
Primary care	24 (14.0)
First hospital unit of admission	
Emergency unit	22 (12.8)
Stroke unit	72 (41.9)
Intensive care unit	28 (16.3)
Neurology unit	20 (11.6)
Internal medicine unit	29 (16.9)
Cardiology unit	1 (0.6)
Imaging test	
Number of imaging tests	163 (94.8)
Type of imaging testing	
CT scan without contrast injection	99 (61.5)
CT scan with contrast injection	38 (23.6)
MRI	61 (37.9)
Cerebral angiography angioscan or angioIRM	13 (8.0)
Cervical Doppler ultrasound examination	59 (37.1)
Thrombolysis treatment	28 (16.3)

a Calculated as the sum of all durations in all units on 182 patients.

Almost all patients (95%) received at least one imaging test, mainly CT scan without contrast injection (61.5%) and 37% of patients had cervical Doppler ultrasound examination. Patients admitted to hospitals were treated within an average of 2±2.6 h and 16.3% received thrombolysis treatment. Length of stay was on average 13.6±9.8 days (Table 3).

According to the GPs, admission to the hospital occurs within 1 h of the event. Length of stay is up to 2 weeks. In the random sample, two of the five GPs were not familiar with rt-PA treatment and could not differentiate it from heparinotherapy. All five agreed that imaging and transfer pathways in hospital units were well described in the hospital letter while clinical scales of severity were not used at hospitals or not reported in the hospital letter. However, scales are used in the rehabilitation centers.

### Follow-up phase

At discharge, 47.6% of patients were transferred to rehabilitation centers, 16.2% to nursing homes, and 31.9% were discharged to their homes (Table 4). Of the latter, 23.7% received specific home medical care. All five GPs estimated that 50% of patients end up at the rehabilitation centers after stroke and estimate that the re-hospitalization rate is 10–15%.

During the 12 months follow-up period, re-hospitalization was confirmed for 21.6% of the patients with average length of stay 15.3±14.2 days. Further, IS patients were assisted by professional carers (53%), personal carers (28.9%), or both (18.1%). Of the employed patients, 59% had at least one reported sick leave and 5.9% changed their occupation.

Additionally, patients sought outpatient medical care by visiting their GPs (11.7 visits) psychologist or psychiatrist (9.5 visits), and neurologists (2.2 visits). Patients received on average 21.1 weeks of nursing care, 17.1 weeks of physiotherapy, 11.0 weeks of occupational therapy, and 12.7 weeks of speech therapy.

### Mortality

In our sample, 24 (13.0%) patients died in the first year following the stroke event for all causes. Of them, 10 occurred in the acute phase and 14 in the chronic phase. Stroke was attributed to 16 of the cases: four were due to the actual stroke event, five were due to stroke recurrence, and seven were due to stroke sequel. According to the GPs, death rate could range between 13 and 20%.

**Table 4:** Healthcare resource utilization during Chronic Phase

	Total, *N=*185 *n* (%)
First place after hospital discharge	
Home	45 (24.3)
Rehabilitation center	88 (47.6)
Nursing home	30 (16.2)
Home medical care	14 (7.6)
Long-term hospitalization	–
Mean length of stay in days	
Home	222.1 (163.72)
Rehabilitation center	32.7 (16.13)
Nursing home	102.9 (137.17)
Home medical care	175.9 (145.2)
Long-term hospitalization	–
Re-hospitalization	40 (21.6)
Re-hospitalization LOS	15.3 (14.2)
Outpatient visits	
Mean number of nursing care visits (weeks)	21.1 (19.2)
Mean number of physiotherapist visits (weeks)	17.1 (18.60)
Mean number of occupational therapy (weeks)	11.0 (10.89)
Mean number of speech therapist	12.7 (11.90)
Mean number of neurologist visits	2.2 (3.1)
Number of general practitioner visits	11.7 (11.9)
Psychological support	9.5 (12.4)
Patient status according to social reimbursement among employed patients	
Sick leave	28 (59.3)
Changed occupation	11 (5.9)
Change to part time job	4 (2.2)
Caregiver	83 (44.9)
Professional	44 (53.0)
Personal	24 (28.9)
Still follow-up by the general practitioner	151 (81.6)

## Discussion

This study identified and recruited a sample of IS patients through GPs in Germany. We described the characteristics of patients and their management during the acute phase, as well as the management and healthcare resource utilization post-discharge during a 12-month follow-up period. GPs usually manage IS patients after hospital discharge, and are well informed about their patients’ acute phase via hospital letters, which describe the hospital stay, procedures performed, and the treatment administered. These attributes qualified GPs to identify and recruit patients and further to collect and report information about the pre-admission phase, during hospital stay, and post-discharge of IS patients.

Patients were recruited in a systematic way and were representative of the general IS patients population and consistent with the literature; our sample had similar demographic distribution in terms of age, sex, and medical history, specifically atrial fibrillation and previous history of stroke (8, 11).

The current DGN and DSG guidelines recommend using CT scan as the main apparatus for diagnosis of stroke and must be performed as soon as possible. Consistent with the DGN and DSG guidelines, almost all patients (94%) received imaging procedures; 85% received CT scans as a diagnostic procedure. In addition, our data are concordant with the published literature; Grau et al. reported 96.4% of patients had an imaging test during hospital stay, and 84.8% had a CT scan or MRI (8).

Length of stay at hospital was 13.6; 95% CI: 12.2–15.0, slightly higher than in the Diagnosis Related Group (11 days) and in published studies (11.1–13.3 days) (10, 12).

Thrombolysis with alteplase is a highly effective evidence-based treatment recommended for IS and should be administered within a 3 h time window after onset of symptoms in patients with acute IS (13). In Germany, alteplase is the only approved thrombolytic for patients with acute IS and the current knowledge about this treatment is still lacking. Generally, not all patients who qualify for this treatment receive it, resulting in lower rates of utilization. A survey by the European Stroke Facilities estimated alteplase rates to be as low as 4.76% of all IS patients in 178 participating hospitals in Germany and Austria (14). This rate was much lower than the estimated optimal rates of alteplase estimated in a community-based study. This study included patients with IS in the Netherlands, and estimated the number of patients eligible for rt-PA therapy if delays between onset and admission to hospital are to be avoided. Delays were defined as when the duration from onset to hospital admission exceeded 1.5 h. Doctor delays were defined as doctors taking more than 20 min to reach the patient. The study estimated that if delays can be avoided, the optimal proportion of acute IS patients to be treated with alteplase, can be as high as 24% in a given area (15). In the present study, the rate of thrombolysis was 16%. This estimation was possibly overly optimistic. Post-survey, GPs received more information about alteplase and were asked to redefine who of their patients received alteplase treatment. The alteplase rate decreased from 16 to 8% but remained substantially higher than the reported rates in the German population (4.7%). The high alteplase rate in this study can be explained by lack of knowledge about the treatment amongst the GPs, who extracted the information, even after having received more educational material about the treatment. Alternatively, the GPs did not differentiate between alteplase and other treatments such as heparinotherapy.

This method had several limitations. First, some GPs may have relied on their memory to recruit patients rather than using their electronic medical records or database to include patients in chronological order, based on the occurrence of stroke as was stated in the study protocol. This may have excluded deceased patients or patients transferred to nursing homes, thus resulting in lower mortality rate (13%) than that in the literature (14.7%) (16). Second, GPs may not have had access to all data elements such as sick leave, which was underreported. This might be because the first sick leave is usually prescribed by the doctors at hospital. Third, although we intended to have a representative sample of the GPs, practices in this study were self-selected and may not be typical of all GP practices in Germany. Physicians may have responded in a specific way out of interest in the management of IS stroke (17) and this may have also inflated the reported results.

## Conclusions

This study describes the management of IS at onset of symptoms, at the acute phase in the hospital and during a 12-month follow-up. We described the healthcare resource utilization of IS patients post-discharge, which is a major driver of economic burden in long-term care. In addition, we documented that GPs receive a hospital letter which details the pathway in the hospital (unit stays) and imaging tests. GPs appear to provide reliable data about patient management. Also, we demonstrated the lack of knowledge among GPs about thrombolysis treatment and the fact that the rate of thrombolyzed patients is still low in Germany.
